# Effect of the COVID-19 Lockdown on Air Pollution in the Ostrava Region

**DOI:** 10.3390/ijerph18168265

**Published:** 2021-08-04

**Authors:** Jan Bitta, Vladislav Svozilík, Aneta Svozilíková Krakovská

**Affiliations:** 1 Meshcheryakov Laboratory of Information Technologies, Joint Institute for Nuclear Research, Moscow Region, 141980 Dubna, Russia; 2 Faculty of Materials Science and Technology, VSB—Technical University of Ostrava, 708 00 Ostrava-Poruba, Czech Republic; 3 Faculty of Mining and Geology, VSB—Technical University of Ostrava, 708 00 Ostrava-Poruba, Czech Republic; krakovska@jinr.ru; 4 Frank Laboratory of Neutron Physics, Joint Institute for Nuclear Research, Moscow Region, 141980 Dubna, Russia

**Keywords:** Ostrava, COVID-19, air pollution, NO_x_, PM_2.5_, cluster analysis, monitoring

## Abstract

A proper estimation of anti-epidemic measures related to the influence of the COVID-19 outbreak on air quality has to deal with filtering out the weather influence on pollution concentrations. The goal of this study was to estimate the effect of anti-epidemic measures at three pollution monitoring stations in the Ostrava region. Meteorological data were clustered into groups with a similar weather pattern, and pollution data were divided into subsets according to weather patterns. Then each subset was evaluated separately. Our estimates showed a 4.1–5.7% decrease in NO_x_ concentrations attributed to lower traffic intensity during the lockdown. The decrease of PM_2.5_ varied more significantly between monitoring stations. The highest decrease (4.7%) was detected at the traffic monitoring station, while there was no decrease detected at the rural monitoring station, which focuses mainly on domestic heating pollution. The key result of the study was the development of an analytical method that is able to take into account the effect of meteorological conditions. The method is much simpler and easy to replicate as an alternative to other published methods.

## 1. Introduction

Ostrava is the third largest city of the Czech Republic. With a population of approximately 300,000 inhabitants, it is the center of a densely inhabited industrial region with more than a million inhabitants, which directly borders Polish Rybnik and Katowice industrial regions. The industrial character of the Upper Silesia region was determined by rich hard-coal deposits, which have been mined here since the 18th century. The presence of coal deposits allowed the growth of industries that use coal as an energy source or feedstock, such as steel production, electricity generation, chemical industry, etc., and downstream industries like machine industry. The presence of employment opportunities also led to the quick population growth during the 19th century and most of the 20th century.

The high population density, combined with the presence of energy and feedstock intensive industries, resulted in serious environmental problems, including air quality, which peaked in the 1970s [1]. There has been an intensive effort to remedy the environmental problems of the Ostrava region, which has brought significant improvements. However, the region remains the most air polluted part of the Czech Republic, and the Czech-Polish Upper Silesian industrial region is one of the most air polluted regions of the European Union.

### 1.1. Air Pollution

Air pollution is caused by the emission of chemical substances into the atmosphere. Pollutants are chemical substances that have an observed negative effect on any part of the environment, i.e., ecosystems, the Earth’s climate, human health or properties. Pollutants can be divided into primary pollutants, which are directly emitted into the atmosphere, and secondary pollutants, which are products of chemical and photochemical reactions in the atmosphere [2,3].

The standard classification divides pollution sources into two groups according to their origin, namely, anthropogenic sources and natural sources. Natural sources of air pollution are natural biotic or abiotic processes such as forest fires, lightning, sea spray, pollen and mold transmission, decomposition processes, etc. Anthropogenic pollution is the result of human activities and can therefore be reduced or prevented. Human activities are concentrated in populated areas, so air pollution is also usually most severe in areas with a high population density, mainly as a result of certain activities, i.e., fuel burning, material processing, energy-intensive processes, etc.

Commonly monitored air pollutants are sulphur dioxide, carbon mono- and dioxide, nitrogen oxides, benzo[a]pyrene, and other polyaromatic hydrocarbons [4]. Their most common source are thermal processes. Nitric oxide (NO) and nitrogen dioxide (NO_2_) are components of nitrogen oxides (NO_x_). Their concentrations in urban areas are currently mainly associated with car traffic [5]. Most NO_x_ emissions consist of NO. In the free atmosphere, NO reacts with oxygen and hydroperoxy radical (HO_2_^•^) in the air into NO_2_.

Particulates are another common air pollutant. Particulate matter (PM_x_) is usually monitored by its diameter fraction: coarse—PM_10_, fine—PM_2.5_ and ultra fine—PM_1_ particles. These fractions are defined as particles with a diameter of ≤10 μm, ≤2.5 μm and ≤1 μm. The physical and chemical composition of particulate pollution is very diverse. PM_x_ consists of a combination of mineral particles, soot, bacteria, pollen, mold, salts, organic materials, etc. [2].

The study focuses on two pollutants. PM_2.5_ is one of the most significant air pollutants in the region. Its sources are highly spatially variable through the Ostrava region. It can represent different kinds of pollution at different sites. NO_x_ were selected to represent car traffic pollution in the region.

### 1.2. Air Pollution Monitoring

The air pollution monitoring network of the Czech Republic is run by the Czech Hydrometeorological Institute (CHMI). The CHMI operates a majority of monitoring stations, collects measurement data from other organisations and publishes them. The legislative norm defining such activities is Act No. 201/2012 Coll. on air protection as amended (‘Air Protection Act’). There are currently 198 air pollution monitoring stations in the Czech Republic, 127 of which are run by the CHMI and 71 stations are run by other governmental organisations and private companies. According to the EU legislation [6], monitored pollutants are PM_10_, PM_2.5_, SO_2_, NO_x_, NO_2_, CO, benzene, benzo[a]pyrene, and toxic metals in aerosol (Pb, Cd, As, Hg). The monitoring network is the densest in regions where increased air pollution occurred historically, including the Ostrava region. In 2019, 12 monitoring stations were operated in Ostrava, and 36 monitoring stations were run in the Ostrava region [7].

The density of the monitoring network enables the selection of specific monitoring stations covering different aspects of air pollution in the region.

### 1.3. Air Quality Management

The city of Ostrava forms part of the Ostrava/Karviná/Frýdek-Místek conurbation, which is the subject of the Czech Environment Ministry’s Air Quality Improvement Program. According to § 9 par. 4 of Act No. 201/2012 Coll. on air protection, the aim is to achieve the required air pollutant levels as soon as possible, and then to maintain and improve air quality throughout the conurbation. Participating organizations must proceed in accordance with legislative requirements. As a participant in the program, Ostrava implements regular short-term air quality improvement programs, incorporating the Action Plan for reducing air pollution within the city. The third update of the Action Plan (2017) is currently being implemented.

On 22 September 2020, a document, which is an updated air quality improvement program for the agglomeration of Ostrava/Karviná/Frýdek-Místek—CZ08A for the period 2020+, was approved. The 2020+ program was preceded by the air quality improvement program for the agglomeration Ostrava/Karviná/Frýdek-Místek—CZ08A of 14 April 2016, file no.:23967/ENV/16, which was issued in accordance with the Air Protection Act as amended on 14 April 2016 in the form of measures of a general nature [8].

The Action Plan of the Statutory City of Ostrava for the Implementation of the Air Quality Improvement Program of the Ostrava/Karviná/Frýdek-Místek Agglomeration - CZ08A contains specific information on activities/projects. It also incorporates a schedule for the implementation of relevant activities and partial steps of individual projects, financial demands, financial coverage, deadlines, and internal responsibilities. In May 2020, on the basis of a contract concluded with the State Environmental Fund of the Czech Republic, a report on the fulfilment of the proposed activities was prepared [9].

On 31 August 2015, the project “Sustainable Mobility Plan Ostrava” was successfully completed. In 2018–2019, a gradual evaluation of the fulfilment of individual tasks of the Action Plans and the fulfilment of individual items of project reservoirs took place. In 2021, work is underway to update the key tasks of the Action Plans. The Sustainable Mobility Plan is a strategic document designed to meet the mobility needs of people and businesses in and around cities, and to ensure a better quality of life. It is a way of tackling transport problems in urban areas more effectively. The aim of the Sustainable Urban Mobility Plan is to create a sustainable urban transport system with at least the following objectives: Ensure that the accessibility offered by the transport system is available to all; Improve transport safety; Reduce air pollution, noise pollution, greenhouse gas emissions and energy consumption; Improve the efficiency and economy of passenger and freight transport; Contribute to improving the attractiveness and quality of the urban environment and urban design [10].

Another CLAIRO project (Clean Air and Climate Adaptation in Ostrava and Other Cities) by the Silesian University in Opava as one of the main partners is implemented by the city of Ostrava. The project aims to systematically reduce air pollution by planting suitable greenery with a proven ability to absorb air pollutants from various sources [11].

### 1.4. COVID-19 Disease

**Figure 1:**
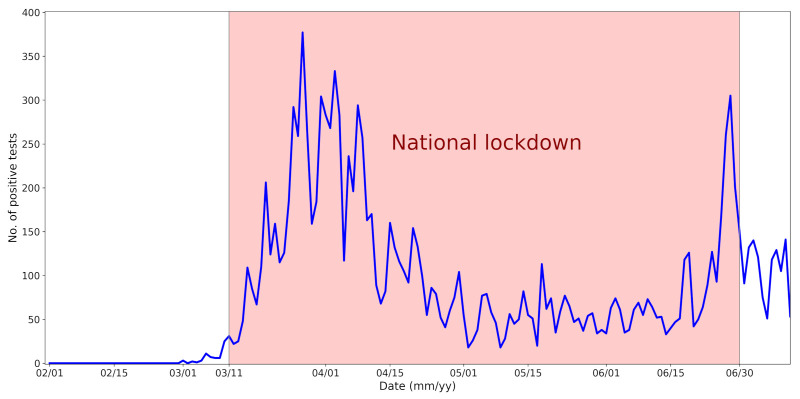
Number of positive COVID-19 tests in the Czech Republic.

In December 2019, a cluster of acute respiratory diseases, now known as novel coronavirus-infected pneumonia, occurred for the first time in the Wuhan district, Hubei Province of the People’s Republic of China [12]. The analysis of samples from affected patients revealed that their symptoms were caused by a coronavirus, later named severe acute respiratory syndrome (SARS) coronavirus (CoV) 2 (SARS-CoV-2) [13]. Coronaviruses belong to Coronaviridae, which is a family of RNA viruses [14]. Within five months, the disease affected more than 210 countries. In March 2020, the World Health Organization announced that the spread was a global pandemic [15]. Due to the high degree of uncertainty about containing the virus, many countries imposed national measures focused on restrictions to day-to-day life. In the Czech Republic, the first three cases of the disease were confirmed on 1 March 2020. The first wave of the epidemic in the Czech Republic culminated around 12 April 2020, when a total of 4750 people infected with COVID-19 were registered (Figure 1), 436 of whom were hospitalized, including about a hundred patients in serious condition. Thereafter, the number of recovered patients began to outweigh the number of newly infected, and the number of hospitalized patients also declined. The number of people in the Czech Republic with a positive test for COVID-19 stabilized at 2000–2500 during May and June 2020 [16].

In the spring of 2020, the national lockdown included the closure of restaurants, nonessential shops, gyms, and swimming pools. Travelling was limited to essential shopping, work purposes, and taking care of close relatives. Some exceptions were allowed, such as going outside for exercise or spending time in nature [17].

### 1.5. COVID-19 Disease Influence on Air Pollution

The situation caused by the spread of the SARS-CoV-2 virus disease and the resulting pandemic significantly affected the social and economic activities of society across the world. A large number of studies is devoted to the influence of air pollution on the spread or consequences of the COVID-19 disease [12].

This unique situation is a suitable moment for the assessment of the influence and spatial-temporal distribution of anthropogenic pollution.

To reduce pandemic consequences, there were applied different restrictions and regulations, such as restrictions on the free movement of persons, medical face masks, restrictions on social activities, restrictions on sports activities, restrictions on trade and services, restrictions on emergency medical care, population testing, and the closure of borders (states, regions, municipalities). The measures could lead to changes in the spatial-temporal distribution of air pollution concentrations. The given changes are presumed to have a mostly local character due to anthropogenic emissions.

There are a number of studies that investigated the dynamics of air pollution in relation to the COVID-19 disease. These studies can be classified according to the analysis used. A significant number of studies focus on comparing the pre-pandemic situation and the pandemic situation when measures and lockdowns were applied [18,19,20,21,22]. This approach is not entirely correct. It is necessary to bear in mind the variability of meteorological conditions.

Another group of studies used more sophisticated methods that incorporated the influence of factors such as meteorological conditions (precipitation, temperature, atmospheric stability, pressure, etc.) [23]. The factor of meteorological conditions is significant, especially in regions with a changeable character of the weather. The study performed by Beloconi et al. [24] presents the results of the Bayesian spatio-temporal (BST) model, which was developed to assess changes in NO_2_ and PM_2.5_ concentrations in Europe. Factors describing land and vegetation cover, impermeability, settlements, terrain, transport, Normalized Difference Vegetation Index (NDVI) and other remote sensing data sources, humidity, meteorological data, and dust were included in the modeling. The research presented by Bekbulat et al. [25] shows an approach that focuses on the comparison of air pollution data describing specific weeks. Pairs for the comparison were chosen based on the similarity of weather conditions during those weeks. The weather situation was taken into account in the study performed by Baldasano [26].

Studies that focused on the effect of the COVID-19 lockdown on air quality did not take meteorological factors into account or used advanced analytical tools that would be highly difficult to replicate on different datasets. A simpler and easily replicable method would be handful in regions where meteorological conditions are highly variable and simple year-over-year comparisons cannot provide proper answers.

## 2. Methods and Data

The late winter and spring weather in Central Europe is highly variable. The region lies between the still cold mass of Scandinavia and the already warm and quickly warming Mediterranean region, between the dry continental air in the east and the humid air of the North sea region. Any weather front, any movement of cyclones and anticyclones can result in a rapid weather change. For this reason, the spring weather can significantly differ from year to year. Any air quality analysis and/or comparison should filter out the weather influence.

Therefore, the analysis was divided into three logical steps:

- Clustering of meteorological data;
- Testing of the pollution difference in each weather cluster;
- Estimation of the lockdown effect on air quality.

All data processing, analysis and graphs in the study were performed in the Python 3.7 programming language. *Pandas* and *numpy* modules were used for data processing, the *scipy* module was used for statistical analysis, the *scikit-learn* module was used for k-means clustering, and the *matlibplot* and *seaborn* modules were used for graph generation. All the modules above are part of Python’s Anaconda distribution [27].

### 2.1. Cluster Analysis

**Figure 2:**
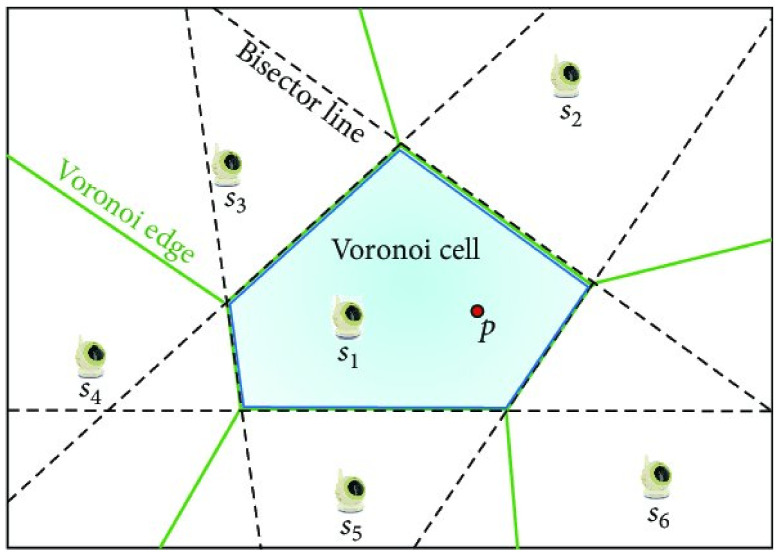
Voronoi cells (CC BY 3.0) [28].

K-means clustering is a well known clustering method. Its goal is to make a partition of *n* data vectors into *k* cluster subsets. Each cluster is represented by a centroid vector, and each data vector is placed to the cluster where the nearest representative centroid is located. The default option of the distance measurement is the Euclidean metric, however, other metrics can also be used. Cluster centroids divide the vector space into Voronoi cells (Figure 2).

K-means clustering is an optimisation problem that involves searching for the positions of cluster centroids to minimize the inertia of the cluster model. Inertia is defined as the sum of distance squares between data vectors and their corresponding cluster centroids.

K-means clustering is known to be an NP-hard problem [29]. There is no computational algorithm that would solve the optimisation problem of k-means in *reasonable* time. However, there are iteration algorithms that enable quick computations to find the local minima of inertia. The global minimum is estimated by running the iteration algorithm several times with different initial approximations and selecting the best performing result [30].

### 2.2. Analysis of Variance Testing

Analysis of Variance (ANOVA) is a class of statistical models designed to analyze the difference between the means of datasets. ANOVA tests compare several datasets with each other and examine whether there are statistically significant differences among their means. A special case of ANOVA tests is the *t*-test, which compares the difference between the means of two datasets [31].

### 2.3. Pollution Monitoring Data

There were two pollutants selected for analysis, i.e., PM_2.5_ and NO_x_. The PM_10_ concentrations were omitted because they highly correlate with PM_2.5_. The correlation between PM_2.5_ and PM_10_ is greater than 0.97 at all analyzed monitoring stations, and the analysis of PM_10_ would not bring new insights. The concentrations of NO_x_ and NO_2_ are also strongly correlated (0.84–0.86) at all analyzed monitoring stations. We selected the NO_x_ for analysis since they better reflect car traffic pollution, mainly at traffic monitoring sites where a large part of NO has not yet reacted with oxygen to form NO_2_.

Other main pollutants (SO_2_, benzo[a]pyrene) that were measured at monitoring stations were not used due to data quality. SO_2_ concentrations are commonly nearby or below the detection limit of monitoring devices, while benzo[a]pyrene concentrations are determined from 6-day samples and the data do not have sufficient time resolution.

**Figure 3:**
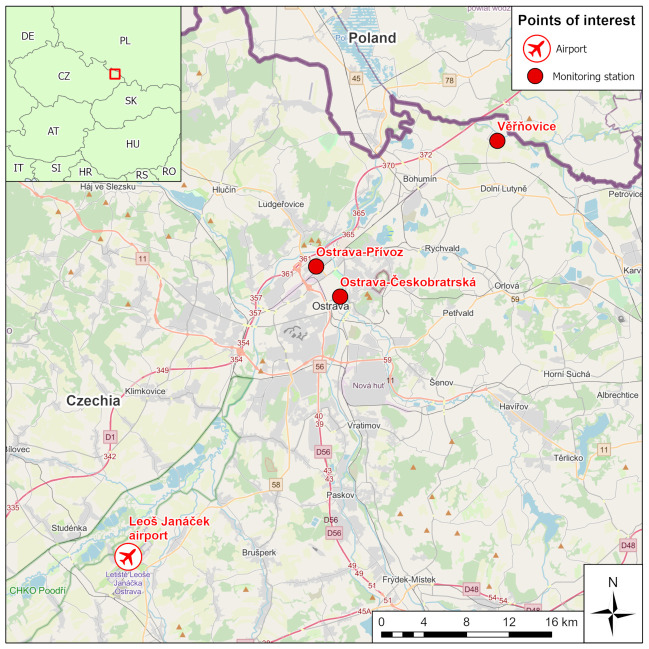
Location of pollution monitoring stations and the Ostrava airport.

Three pollution monitoring stations in the Ostrava region were selected for analysis. Each station represents a different source of air pollution (Figure 3, Table 1). A more detailed description of the selected monitoring stations is available in Appendix A.

All three selected stations measured both PM_2.5_ and NO_x_ concentrations. All monitoring stations measured the pollutants at 1 h intervals. Hourly data for 2020 were not yet published, but were kindly provided for the study by the Czech Hydrometeorological Institute. The hourly data were used to calculate daily averages, which were later utilized in the study. The brief exploratory statistics of pollution data are shown in Table 2.

### 2.4. Meteorological Data

**Figure 4:**
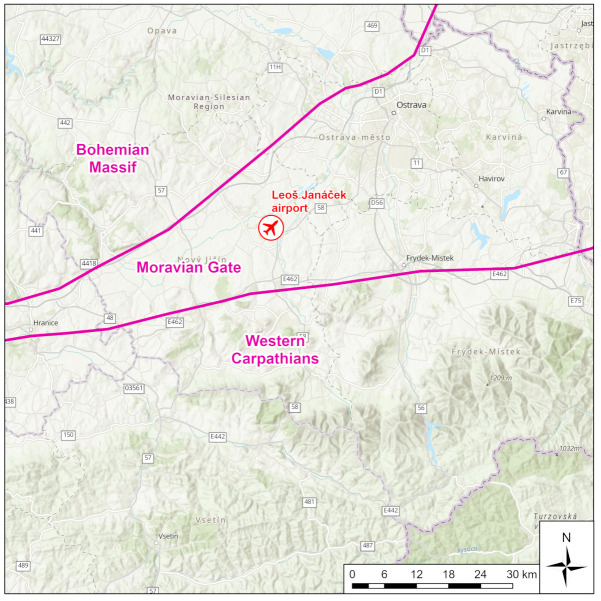
Moravian Gate, Basemap: ESRI Topographic Map.

Meteorological data used in this study were measured at the meteorological station of the Leoš Janáček Airport Ostrava (LKMT). The meteorological station is located in the open field of the airport and is not affected by local conditions (e.g., buildings in the vicinity). Therefore, the station well represents general meteorological conditions in the wide valley of the Moravian Gate, which also includes Ostrava (Figure 4).

**Figure 5:**
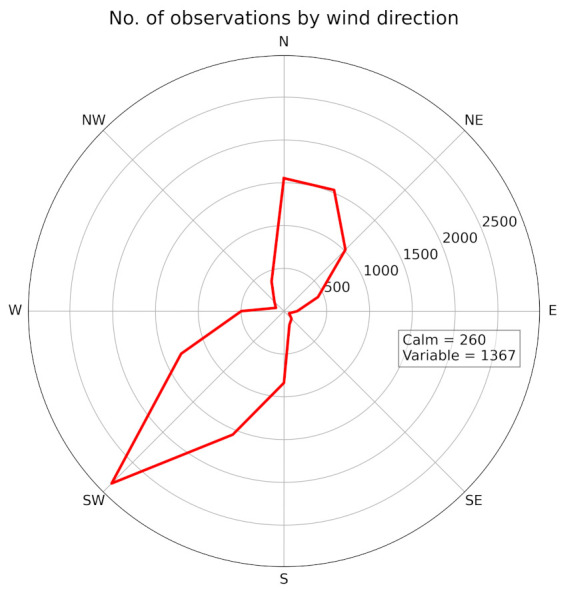
Number of observations by wind direction.

The data were downloaded from the Weather Underground website [32] as a set of measurements covering the whole time period of the study. Measurements were taken at 30 min intervals. The time period is from 1 February 2019 to 30 June 2019 and from 1 February 2020 to 30 June 2020. 14,352 meteorological measurements were processed. Each measurement consisted of date, time, temperature, humidity, wind direction, wind speed, and atmospheric pressure. All meteorological variables were used to calculate daily averages. The only exception was the wind speed data, in which the fractions of each wind direction category were calculated. The wind direction data were categorized into 18 categories. There were 16 categories representing wind direction. The remaining two categories were calm wind and variable wind. The brief exploratory statistics are shown in Table 3 and Figure 5 (Numerical values of the wind direction statistics are available in Appendix B).

The day-of-year variable was also included in the meteorological data. This variable represented the intensity of insolation, which changes rapidly in late winter and spring around 50N latitude, at which the study was performed.

## 3. Results

### 3.1. Meteorological Data Clustering

The meteorological data were grouped using the k-means algorithm into clusters with similar weather conditions. During the process, two issues had to be solved:

- Data normalization;
- Number of clusters;

The data needed to be normalized. Without the normalization, clustering would be disproportionally weighted towards the parameters with the highest numerical differences. A uniform normalization was selected. Each meteorological parameter was linearly transformed into the [0,1] interval by the formula:

$$value_{transformed}=\frac{value_{original}-value_{min}}{value_{max}-value_{min}}$$

The most suitable number of clusters was determined by the experiment. There was clustering performed on the meteorological data for the number of clusters ranging from 2 to 40. The optimal number of clusters was selected by the analysis of the inertia of cluster models.

**Figure 6:**
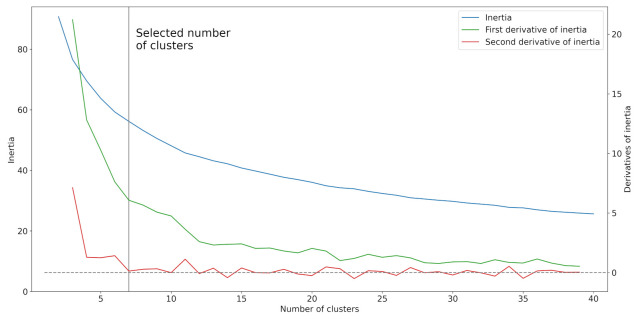
Dependence of k-means inertia on the number of clusters.

The first derivative of the function *I*, which captured the dependence of inertia on the number of clusters (visualized in the graph above Figure 6), was approximated by the central difference formula:

$$I^{\prime }\left(n\right)\approx \frac{I(n-1)-I(n+1)}{2}$$

We decided to swap the order of the *I* function values in the formula to get positive values of the first derivative.

The second derivative of the function *I* was approximated by the following difference formula:

$$I^{\prime \prime }\left(n\right)\approx I\left(n-1\right)-2I\left(n\right)+I\left(n+1\right)$$

According to the second derivative approximation, the function *I* can be split into two parts. In the first part, the function shows a significant improvement in clustering performance. The second part of the function shows only a slight linear improvement in clustering performance. This is the reason why we selected the split value 7 as the optimal number of clusters for k-means clustering. The centroids of each cluster are shown in Table 4.

### 3.2. Variance Analysis of Pollution Data

**Figure 7:**
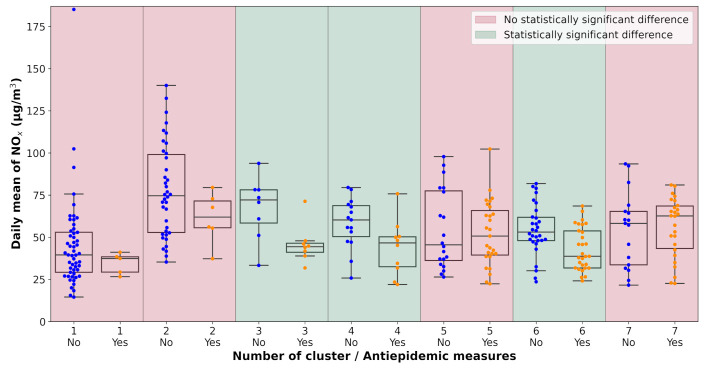
Results of the ANOVA test, Ostrava-Českobratrská, NO_x_.

**Figure 8:**
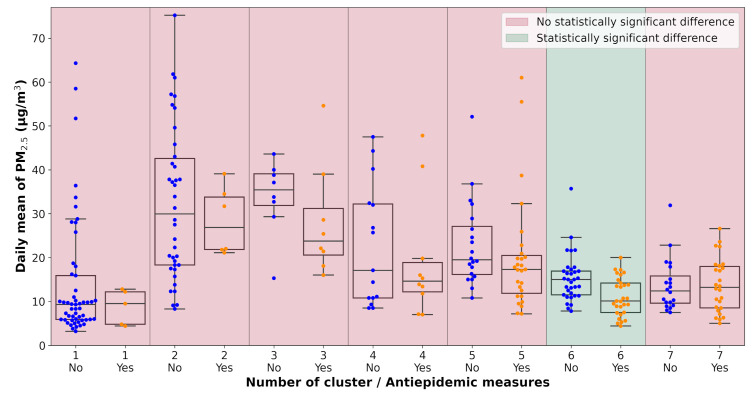
Results of the ANOVA test, Ostrava-Českobratrská, PM_2.5_.

**Figure 9:**
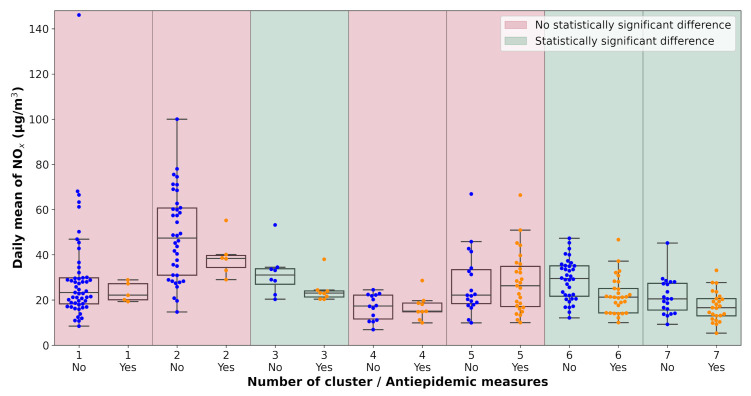
Results of the ANOVA test, Ostrava-Přívoz, NO_x_.

**Figure 10:**
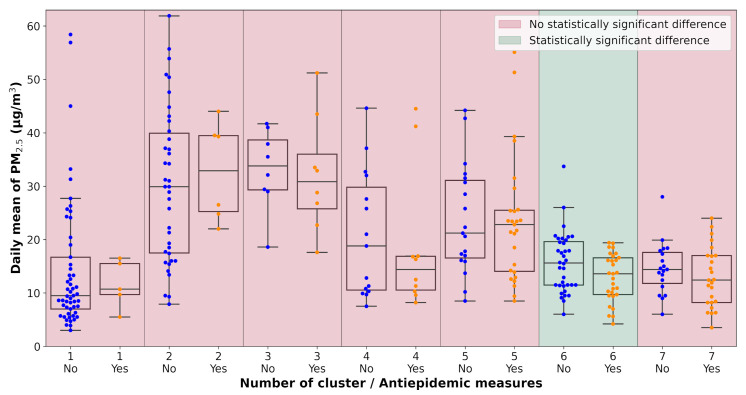
Results of the ANOVA test, Ostrava-Přívoz, PM_2.5_.

**Figure 11:**
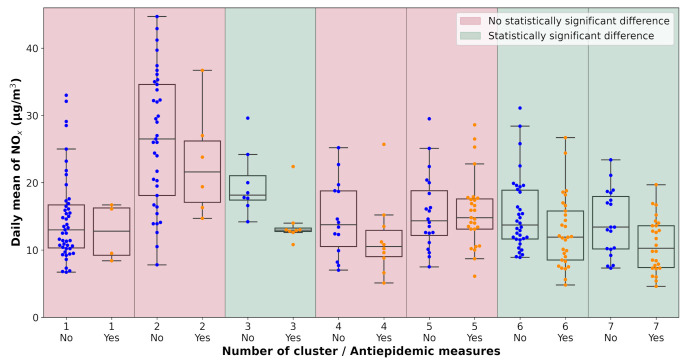
Results of the ANOVA test, Věřňovice, NO_x_.

**Figure 12:**
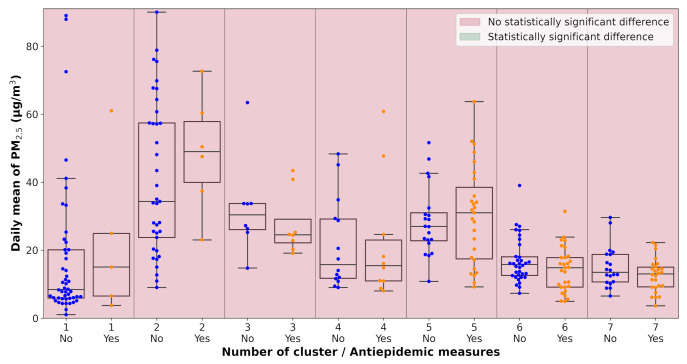
Results of the ANOVA test, Věřňovice, PM_2.5_.

All pollution measurements used in the study were categorized according to the weather cluster they belong to. The next step in the analysis was one-way ANOVA tests performed on each unique dataset defined by a combination of pollutant, monitoring station, and weather cluster. The data subsets were tested for the significance of the lockdown occurrence with α=0.1. The results of the analysis are visualized in Table A2 and Figure 7, Figure 8, Figure 9, Figure 10, Figure 11 and Figure 12. Tabular data with the test results are available in Appendix C.

### 3.3. Lockdown Effect on Air Pollution Estimation

The ANOVA test results were used to estimate pollution concentrations if no lockdown was put into action. Each measurement can be categorized by a unique combination of monitoring station, measured pollutant, and weather cluster. If the ANOVA test found no significant difference for such a category, the measurement remained intact. If the ANOVA test found a significant difference, the measurement was multiplied by the division of the mean value out of lockdown and the mean value in lockdown. The change in the mean value for the February-June period is summarized in Table 5:

## 4. Discussion

There is a significant influence of weather conditions on air pollution, which needs to be accounted when comparing pollution monitoring results. The authors of the study decided to deal with this issue by dividing weather conditions into naturally occurring clusters of similar weather conditions. The k-means algorithm detected 7 such clusters from the meteorological dataset from 1 February 2019 to 30 June 2019 and from 1 February 2020 to 30 June 2020.

Each day of the dataset was labeled by the k-means model, and the labelling was used to divide the NO_x_ and PM_2.5_ pollution data into 7 datasets. Each dataset was tested whether there exists a significant difference between the concentrations measured during the active anti-epidemic in the first wave of the COVID-19 lockdown and the concentrations measured during ordinary social and economic activities. The analysis results made it possible to calculate an estimate of the pollution difference caused by anti-epidemic measures.

The results showed a 4.1–6.7% decrease in the NO_x_ concentrations, probably mainly due to lower traffic intensity. The decrease of PM_2.5_ was not significant at all monitoring sites. The highest decrease was estimated at the Ostrava-Českobratrská traffic station (4.7%). The Věřňovice monitoring station, which mainly focuses on domestic heating pollution, showed no significant effect of anti-epidemic measures on the PM_2.5_ concentrations.

The results of the study are consistent with the results of the study [24], which assessed air pollution throughout the European Union. The article also found a more significant decline in NO_2_ pollution and a lower decline of PM_2.5_.

The study [19] found a higher decline in air pollution. The steeper decline can be attributed to the fact that India is a developing country with a lower level of pollution prevention. This means that any decline in economic and social activities has a greater effect on overall pollution.

The study [20] found a 16% decrease of NO_2_ and a 19% decrease of PM_2.5_ in California when compared with the monitoring results over the previous 5-year period. The effect of weather conditions was not filtered out in this study. Therefore, it is not clear how much of the decline can be attributed to weather conditions and how much can be attributed to anti-epidemic measures.

The study [25] compared air pollution monitoring results across the USA. The study also found a slight decline in air pollution during the COVID-19 lockdown.

### Policy Implications

There are several studies that analyzed air quality in the Ostrava region [5,8,9,10], and their results were used as foundations for policy recommendations, which were gradually implemented. Those studies were based on air pollution dispersion modeling using various models. The COVID-19 lockdown made it possible to study real world effects of emission changes, which is a way to verify model results.

All air pollution studies consider car traffic to be the main source of NO_x_ and NO_2_ pollution in urban areas of the Ostrava region. This result was verified by our study since the decline of NO_x_ at all monitoring sites coincides with the decline in car traffic during the lockdown, when most of the population switched to home office work schedules. Another key source of NO_x_ pollution, industrial sources (electricity production, heat production, steel making), continued their production almost uninterrupted.

The analysis of PM_2.5_ pollution confirmed the importance of air pollution from domestic heating, which is the main source of PM_2.5_ in the region. The greatest decline in the PM_2.5_ concentrations was observed at the Ostrava-Českobratrská traffic station, which can be attributed to the decline in car traffic. No decline in the PM_2.5_ concentrations at the Věřňovice station well coincides with the fact that domestic heating was not affected by the COVID-19 lockdown.

The verification of air pollution dispersion models further strengthens arguments for measures that are currently adopted to reduce emissions from domestic heating and car transport. There is a program run by the Moravian-Silesian regional government, which stimulates the replacement of old stoves using solid fuels by low-emission or emission-free substitutes. The program is aimed at the financing of replacements via direct subsidies and cheap financing [9].

The results of our study also confirm the importance of all measures taken to lower emissions from car traffic. This has been implemented in several programs aimed at traffic intensity reduction (support of public transport, cycling, ride-sharing, etc.), as well as a long-term switch to low-emission or emission-free vehicles. For example, public transport in Ostrava is now mostly electrified (trains, trams, trolleybuses, electric buses), while the rest have switched to low-emission CNG fueled buses [10].

## 5. Conclusions

We managed to develop an algorithm that filters out the effect of weather conditions on pollution concentrations to analyze pairs of datasets coming from the same monitoring station. The algorithm was applied to compare data measured during the active anti-epidemic in the first wave of the COVID-19 lockdown and during ordinary social and economic activities. However, the algorithm is generally applicable on different situations.

The algorithm itself is the main result provided by the study since it allows taking weather conditions into account, while being much simpler and easier to apply when compared to other published methods.

The concentration estimates without the effect of anti-epidemic measures can be further improved by using a more advanced estimation technique applied to each data subset, which is defined by meteorological data clustering. Multidimensional statistical regression or artificial neural network estimates can be applied.

The results of the study confirm the results of air quality studies based on air pollution dispersion modeling and support recommended policies focused on lowering emissions from domestic heating and car traffic. 

## Figures and Tables

**Table 1:** Categorization of monitoring stations.

Monitoring Station	Categorization
Ostrava-Českobratrská	Traffic/Urban/Commercial-Residental
Ostrava-Přívoz	Industrial/Urban/Industrial-Residental
Věřňovice	Background/Rural/Residental-Agricultural

**Table 2:** Statistical summary of the PM_2.5_ and NO_x_ daily averages (μg/m^3^).

	O.-Českobratrská		O.-Přívoz		Věřňovice	
	PM_2.5_	NO_x_	PM_2.5_	NO_x_	PM_2.5_	NO_x_
Minimum	3.2	14.5	3.0	5.3	1.0	4.6
Average	19.1	54.3	19.6	28.5	23.3	16.1
Maximum	75.2	185.1	61.9	146.1	90.0	44.7
Standard deviation	13.4	23.2	12.1	16.5	17.5	7.6

**Table 3:** Statistical summary of meteorological variables.

	Temperature	Humidity	Pressure	Wind Speed
	(deg C)	(%)	(hPa)	(km/h)
Minimum	−11	14	959.3	0
Average	10.2	70.2	985.5	13.8
Maximum	33	100	1012.7	59
Standard deviation	7.3	18.8	7.6	8.6

**Table 4:** Centroid values of k-means clustering.

Cluster	Temperature	Humidity	Pressure	Date	Wind Speed	Wind Direction
	deg C	%	hPa	dd.mm	km/h	-
1	6.5	69	980	26.02.	22.2	SW
2	3.9	74	994	02.03.	10.7	SW and Variable
3	5.4	56	995	05.04.	17.6	NE
4	6.4	75	988	12.04.	17.5	N to NE
5	10.5	56	987	16.04.	10.7	Variable
6	16.2	75	981	25.05.	10.4	SW and Variable
7	16.3	78	987	08.06.	11.2	N to NE

**Table 5:** Summary results of the mean value change.

Monitoring Station	Pollutant	Mean (μg/m^3^)		Change
		Measured	Calculated	(%)
O.-Českobratrská	NO_x_	49.17	51.27	−4.1
	PM_2.5_	15.40	16.15	−4.7
O.-Přívoz	NO_x_	24.49	26.24	−6.7
	PM_2.5_	17.18	17.72	−3.0
Věřňovice	NO_x_	13.86	14.70	−5.7
	PM_2.5_	20.44	20.44	−0.0

**Figure A1:**
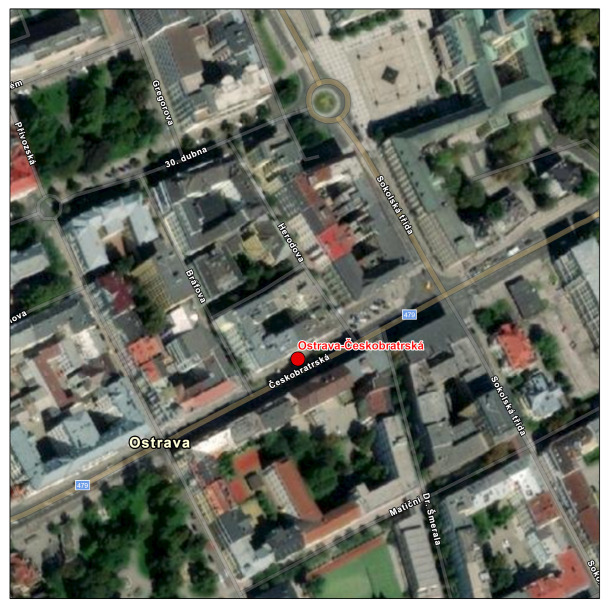
Location of the Ostrava-Českobratrská station.

**Figure A2:**
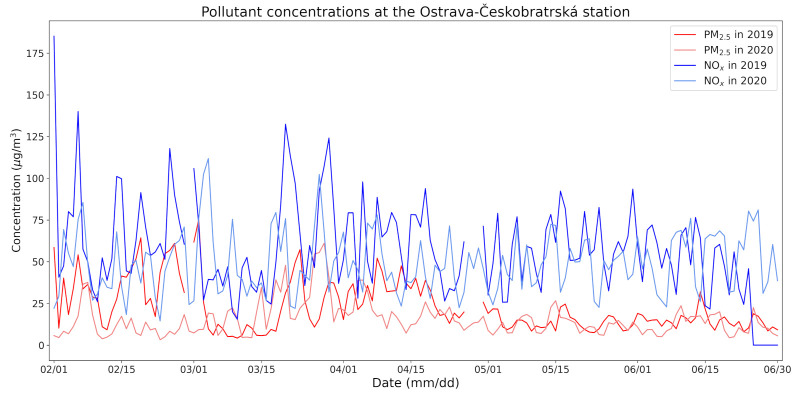
Daily average concentrations at the Ostrava-Českobratrská station.

**Figure A3:**
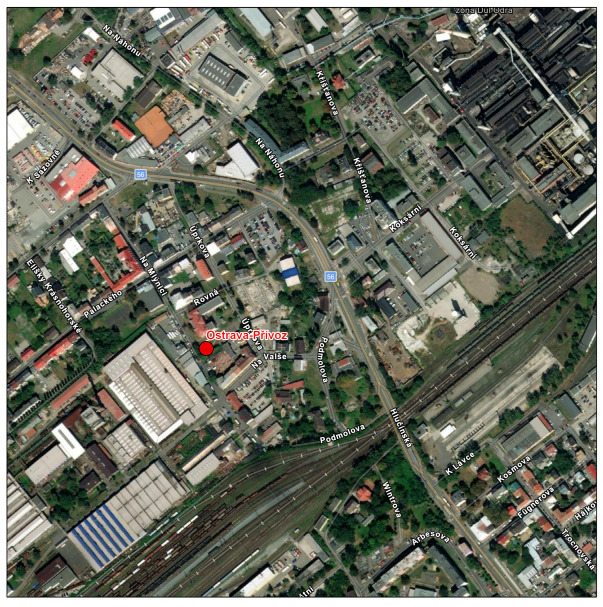
Location of the Ostrava-Přívoz station.

**Figure A4:**
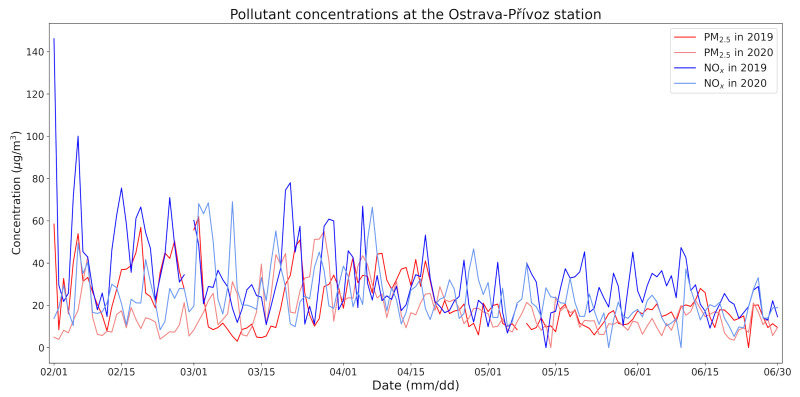
Daily average concentrations at the Ostrava-Přívoz station.

**Figure A5:**
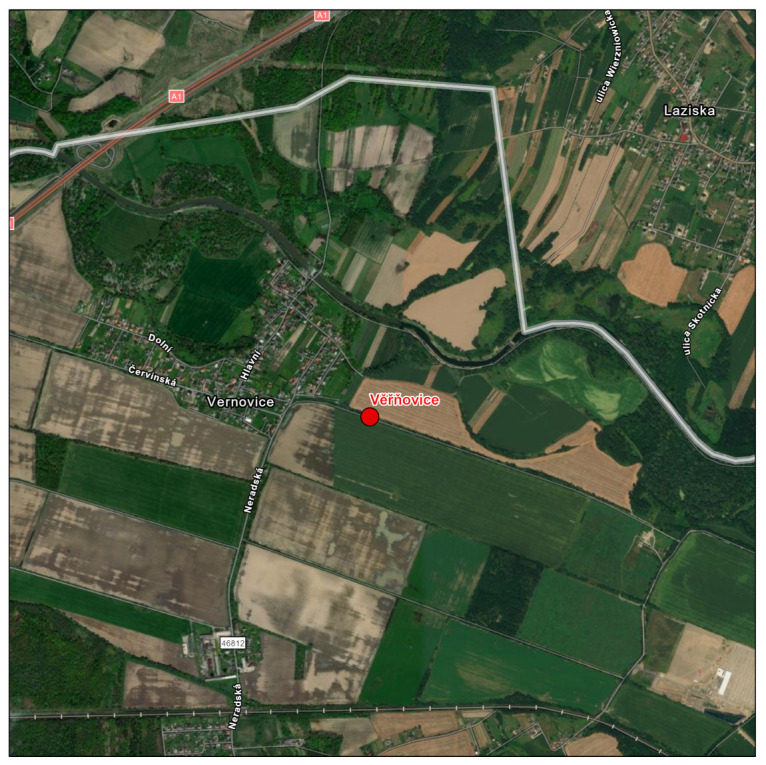
Location of the Věřňovice station.

**Figure A6:**
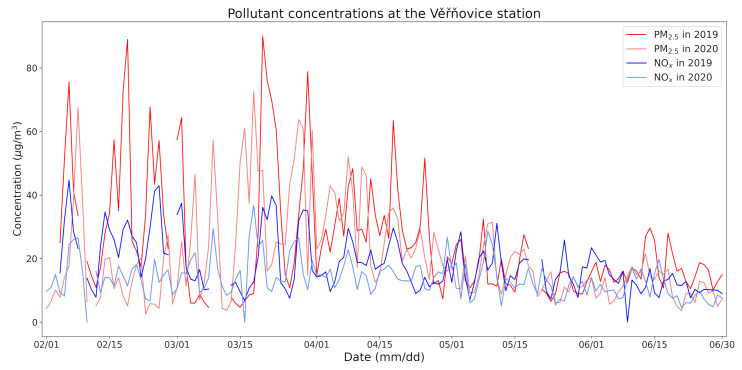
Daily average concentrations at the Věřňovice station.

## Data Availability

All data are freely available at the authors of the study. If you are interested in this data, please contact the authors.

## Appendix A. Pollution Monitoring Stations

### Appendix A.1. Monitoring Station Ostrava-čEskobratrská

The Ostrava-Českobratrská station is located in the street canyon of Českobratrská street in the center of Ostrava. The station is designed to be a traffic hot-spot station and was selected because their data well represent car traffic pollution. Traffic intensity in Českobratrská street was 19,081 cars, 1645 trucks and buses and 49 motocycles during the last published traffic survey in 2016 [33]. Traffic in Českobratrská street is regulated by traffic lights, which results in increased emissions from traffic due to the start-stop behaviour of traffic flow.

### Appendix A.2. Monitoring Station Ostrava-PříVoz

The Ostrava-Přívoz station is located in a schoolyard in the industrial suburb of Ostrava with the mixed residental and industrial development. There is frequented Hlučínská street about 200 m east of the station. Traffic intensity in Hlučínská street was 13,814 cars, 2022 trucks and buses and 64 motocycles during the last published traffic survey in 2016 [33].

### Appendix A.3. Monitoring Station VěřňOvice

The Věřňovice station is located approximately 15 km northeast of the center of Ostrava, near the village of Věřňovice at the Czech-Polish border. The station was chosen to represent pollution from domestic heating and transboundary pollution from Poland, where Polish domestic heating is the dominant source of air pollution in the region [5]. The Věřňovice pollution monitoring station was the site of the highest average annual concentrations of PM_10_ and PM_2.5_ in the Czech Republic in 2019 [7].

## Appendix B. Wind Direction Statistics

**Table A1:** Frequency of wind direction observations.

Wind Direction	No. of Observations
N	1553
NNE	1532
NE	1016
ENE	431
E	155
ESE	66
SE	126
SSE	170
S	838
SSW	1561
SW	2846
WSW	1299
W	498
WNW	101
NW	157
NNW	376
Calm	260
Variable	1367
Total	14,352

## Appendix C. Detailed Results of Anova Analysis

**Table A2:** Results of ANOVA tests.

Monitoring Station	Pollutant	Cluster	Out of Lockdown		In Lockdown		F	*p*	Test Result
			Mean	Count	Mean	Count			
O.-Českobratrská	NO_x_	1	45.00	53	34.56	5	0.756	0.388	No difference
		2	77.59	38	61.47	6	1.897	0.176	No difference
		3	67.45	8	45.81	8	7.790	0.014	Difference
		4	58.25	15	43.75	10	5.236	0.032	Difference
		5	54.32	20	52.25	27	0.111	0.741	No difference
		6	54.48	30	43.07	29	9.725	0.003	Difference
		7	54.60	17	55.99	26	0.051	0.823	No difference
	PM_2.5_	1	13.97	53	8.74	5	0.703	0.405	No difference
		2	32.32	38	28.37	6	0.292	0.592	No difference
		3	33.83	8	28.15	8	1.067	0.319	No difference
		4	22.63	15	19.29	10	0.348	0.561	No difference
		5	22.95	20	20.09	27	0.669	0.418	No difference
		6	15.29	33	11.12	29	10.922	0.002	Difference
		7	13.73	20	13.72	26	0.000	0.997	No difference
O.-Přívoz	NO_x_	1	29.42	53	23.52	5	0.368	0.547	No difference
		2	48.28	38	39.03	6	1.270	0.266	No difference
		3	31.81	8	24.33	8	3.336	0.089	Difference
		4	16.94	14	16.74	9	0.006	0.937	No difference
		5	27.23	19	27.74	27	0.015	0.902	No difference
		6	28.98	36	22.08	29	10.171	0.002	Difference
		7	21.68	20	17.23	25	4.031	0.051	Difference
	PM_2.5_	1	14.35	53	11.58	5	0.246	0.622	No difference
		2	30.16	38	32.68	6	0.170	0.682	No difference
		3	33.15	8	32.13	8	0.047	0.831	No difference
		4	20.79	15	18.76	10	0.163	0.690	No difference
		5	23.71	19	23.38	27	0.010	0.922	No difference
		6	15.67	36	12.89	29	4.683	0.034	Difference
		7	14.67	19	12.96	25	1.115	0.297	No difference
Věřňovice	NO_x_	1	14.69	47	12.68	4	0.365	0.549	No difference
		2	26.42	37	22.98	6	0.650	0.425	No difference
		3	19.83	8	13.94	8	7.627	0.015	Difference
		4	14.62	14	11.67	10	1.562	0.225	No difference
		5	15.56	20	15.69	27	0.006	0.939	No difference
		6	15.32	34	12.67	29	3.723	0.058	Difference
		7	14.04	19	10.80	26	5.985	0.019	Difference
	PM_2.5_	1	17.20	48	22.22	5	0.270	0.606	No difference
		2	41.14	37	48.53	6	0.583	0.449	No difference
		3	32.23	8	27.56	8	0.611	0.448	No difference
		4	21.69	14	22.06	10	0.003	0.955	No difference
		5	28.57	20	30.13	27	0.169	0.683	No difference
		6	16.85	34	14.29	29	2.364	0.129	No difference
		7	15.05	20	12.56	26	2.436	0.126	No difference

## Abbreviations

The following abbreviations are used in this manuscript:

COVID-19	Coronavirus disease 2019
SARS-CoV-2	Severe acute respiratory syndrome coronavirus 2
PM_10_	Particulate matter of size ≤10 μm
PM_2.5_	Particulate matter of size ≤2.5 μm
PM_1_	Particulate matter of size ≤1 μm
CHMI	Czech Hydrometeorological Institute
ANOVA	Analysis of Variance
